# Differences of spinal cord gadolinium enhancement features of neuromyelitis optica spectrum disorder and long-segment degenerative cervical myelopathy

**DOI:** 10.3389/fneur.2023.1191761

**Published:** 2023-07-05

**Authors:** Xingwen Sun, Qiang Zhao, Lihua Zhang, Huishu Yuan

**Affiliations:** Department of Radiology, Peking University Third Hospital, Beijing, China

**Keywords:** neuromyelitis optica spectrum disorder, degenerative cervical myelopathy, magnetic resonance imaging, intramedullary gadolinium enhancement, cervical spine

## Abstract

**Objectives:**

Neuromyelitis optica spectrum disorder (NMOSD) and long-segment degenerative cervical myelopathy (DCM) may have a similar appearance on MRI. This study aimed to identify the differences in spinal cord gadolinium enhancement features between NMOSD and long-segment DCM.

**Methods:**

Spinal cord gadolinium enhancement of 27 NMOSD patients and 30 long-segment DCM patients were retrospectively analyzed. Enhancements were evaluated for their number, length, location on the sagittal images, distribution on the axial images, and form on the sagittal images. The Wilcoxon rank sum test was performed to compare numerical variables. The Pearson chi-squared test was performed to compare categorical variables.

**Results:**

The median number of enhanced lesions (*p* < 0.05), the median length of the enhancements (*p* < 0.05), and the location of enhancement on sagittal images (*p* < 0.05) of NMOSD patients and long-segment DCM patients showed significant differences. The axial distribution of enhancements did not show a significant difference between NMOSD and long-segment DCM patients (*p* = 0.115). On the sagittal images, linear and ring-formed enhancements were observed in 10 (27.0%) and 17 (63.0%) NMOSD patients, respectively. The enhancements in long-segment DCM patients had a transverse band or pancake-like appearance in 15 (50%) patients and an irregular flake-like appearance with a longitudinally oriented long axis in 15 patients (50%).

**Conclusion:**

By analyzing the number, length, location, and form of the gadolinium enhancements, NMOSD and long-segment DCM could be well-differentiated.

## Introduction

Neuromyelitis optica spectrum disorder (NMOSD) is an autoimmune disorder that typically presents with acute transverse myelitis and/or optic neuritis attacks (1). Clinical symptoms of acute transverse myelitis overlap with degenerative cervical myelopathy (DCM), which is the leading cause of cord injury (2). MRI imaging is crucial for the diagnosis of both NMOSD and DCM. Multi-level cervical spondylosis may cause multi-level cord edema presenting as hyperintensity extending over multiple vertebral segments on T2-weighted images, which is similar to NMOSD (1, 3, 4). The misdiagnosis of cervical spondylosis as transverse myelitis has been reported in literature (5, 6). In the case of severe DCM, delayed surgical intervention may worsen the outcome (7). Patterns of enhancement are important clues in differentiating inflammatory conditions from DCM (5, 8). Ozawa et al. have reported that a pancake-like enhancement at or right below the level of maximum compression has high specificity for DCM (9). In their study, the median number of the involved vertebral segments is two (9). Studies focusing on the pattern of enhancement of long-segment DCM caused by multi-level cervical spondylosis involving three or more vertebral segments are scarce. In addition, studies describing the patterns of enhancement of the spinal cord lesions in NMOSD patients are of a small case number (4, 8). In daily practice, we encountered variable appearances of spinal cord enhancements in long-segment DCM and NMOSD patients, which have been rarely reported. Our study aims to systemically analyze the difference in spinal cord enhancement between NMOSD and long-segment DCM caused by multi-level cervical spondylosis.

## Patients and methods

### Patients

This study has been approved by the institutional ethics review board. Informed consent was waived. We retrospectively reviewed patients who underwent cervical spine MRI with gadolinium enhancement from January 2017 to August 2021. The inclusion criteria for NMOSD patients were as follows: (1) serology positive for aquaporin 4 (AQP4) antibody, (2) the presence of longitudinally extensive transverse myelitis lesions (LETM) on MRI, and (3) the presence of gadolinium-enhanced lesion. The inclusion criteria for long-segment DCM patients were as follows: (1) the diagnosis of DCM was confirmed by clinical and radiological evidence and underwent decompression surgery after an MRI exam, (2) the presence of long-segment cervical myelopathy with T2-weighted hyperintense signal extending over three vertebra body segments, and (3) the presence of gadolinium-enhanced lesions. Post-operative MRIs with metal implants in place were excluded to avoid interference caused by metal artifacts.

### Neuroimaging

MRI was performed using a 3T scanner (Discovery MR750, GE Healthcare) with the patient in a supine position. Sagittal and axial T2-weighted and T1-weighted imaging with and without gadolinium-enhancing images were reviewed.

### Imaging assessment

Two radiologists specialized in spinal cord diseases (L. Z. with 13 years clinical experience and X. S. with 3 years clinical experience) reviewed all images. Both radiologists were blinded to the final diagnosis during radiological evaluation. The following features of enhancements were assessed: (1) the number of discrete-enhanced lesions, (2) the total length of the enhanced lesion(s) of every patient as measured by the number of vertebral body segments on the sagittal images, (3) the distribution of the enhancement on the axial images which was described as centric, circumferential, or focal, (4) the distribution of the enhancement in white and/or gray matter, and (5) the form of the enhancement on the sagittal images.

### Statistical analyses

A commercially available software package (SPSS, version 26.0; IBM) was used for statistical analysis. In descriptive statistics, numerical variables were reported in median and range, and categorical variables were reported in number and percentage. The Wilcoxon rank sum test was performed to compare numerical variables, and the Pearson chi-squared test or Fisher exact test was performed to compare categorical variables. A *p*-value of < 0.05 was considered to be statistically significant for all tests.

## Results

A total of 27 NMOSD patients (63% female) and 30 long-segment DCM patients with multi-level cord edema (13.3% female) were recruited (*p* < 0.05). The median ages of NMOSD patients and long-segment DCM patients were 47 years (ranging from 19 to 80) and 51.5 years (ranging from 35 to 65), respectively, (*p* = 0.07). Table 1 summarized the demographic characteristics and clinical features of recruited patients. In NMOSD patients, the T2-weighted hyperintense lesions extended over a median number of four (ranging from 3 to 12) vertebral segments. In long-segment DCM patients, the T2-weighted hyperintense lesions extended over a median number of three (ranging from 3 to 8) segments. All enhanced lesions were found within the T2-weighted hyperintense regions.

**Table 1:** Summary of demographic characteristics and clinical symptoms.

	**NMOSD**	**Long-segment DCM**
Total	27	30
Median age (range)	47 (19–80)	51.5 (35–65)
Sex ratio M:F	10:17	26:4
**Symptoms (no./%)**		
Weakness	20 (74.1%)	17 (56.7%)
Numbness	25 (92.6%)	23 (76.7%)
Paresthesia	12 (44.4%)	9 (30%)
Pain	21 (77.8%)	29 (96.7%)

NMOSD, Neuromyelitis optica spectrum disorder; DCM, Degenerative cervical myelopathy.

**Figure 1:**
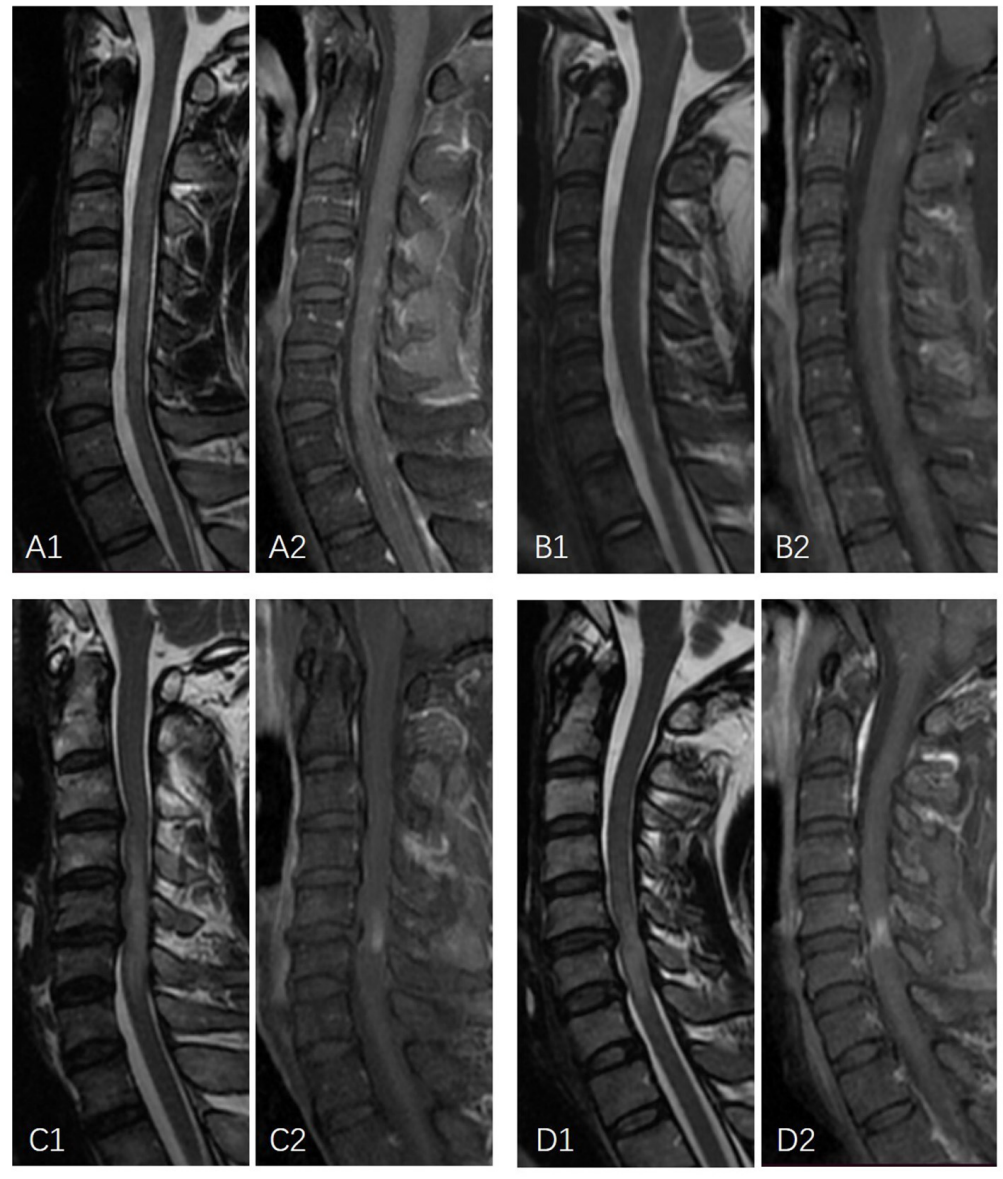
Examples of linear enhancement in NMOSD patients and longitudinally oriented flake-formed enhancement in long-segment DCM. **(A1, A2)** A 26-year-old male patient with NMOSD. Sagittal MRI demonstrates linear T2-weighted hyperintensity with discontinued linear gadolinium enhancement. **(B1, B2)** A 19-year-old female patient with NMOSD. Sagittal MRI demonstrates linear mildly elevated T2 signal intensity with linear gadolinium enhancement. **(C1, C2)** A 46-year-old male with T2-weighted hyperintensity extending over multiple vertebral segments with a longitudinally oriented enhancement flake-like appearance. **(D1, D2)** A 44-year-old male with T2-weighted hyperintensity extending over multiple vertebral segments with irregular flake-appearing enhancement with a longitudinally oriented long axis.

Comparisons of enhancement features between NMOSD and long-segment DCM were summarized in Table 2. The median number of enhanced lesions was 2 (ranging from 1 to 12) in NMOSD patients and 1 (range = 1–1) in long-segment DCM patients (*p* < 0.05). The median length of the enhancement was 3 (ranging from 1 to 7) vertebral segments in NMOSD patients and 1 (range = 1–1) vertebral segment in DCM patients (*p* < 0.05). In total, 3 (11.1%) NMOSD patients had only one enhanced lesion and were limited to one vertebral segment. NMOSD patients with multiple-enhanced lesions often showed a scattered distribution with variable locations on sagittal images. In long-segment DCM patients, the enhancements were located one level above the maximum narrowing in 1 case (3.3%) and at or immediately below the maximum narrowing in 29 cases (96.7%). The enhancements were most frequently located at the C4-5 (8 cases) and C5-6 (18 cases) levels and were all limited to one intervertebral level. The enhancements were central in 8 (28.6%) NMOSD patients and 10 (33.3%) long-segment DCM patients, circumferential in 0 (0%) NMOSD patients and 4 (13.3%) long-segment DCM patients, and focal in 19 (71.4%) NMOSD patients and 16 (53.3%) long-segment DCM patients (*p* = 0.115). In NMOSD patients, the enhancement involves only white matter in 9 (33.3%) NMOSD patients and 6 (20%) long-segment DCM patients. The enhancement involves both gray and white matter in 18 (66.7%) NMOSD patients and 24 (80%) long-segment DCM patients (*p* = 0.99). On the sagittal images, linear and patchy enhancements were observed in 9 (33.3%) and 18 (66.7%) NMOSD patients, respectively (Figure 1). All linear enhancements are located within the white matter with six cases within the posterior column and four cases within the anterior column. The enhancements in long-segment DCM patients had a transverse band or pancake-like appearance in 15 (50%) patients and an irregular flake-like appearance with a longitudinally oriented long axis in 15 patients (50%) (Figure 1).

**Table 2:** Comparisons of enhancement features between NMOSD and long-segment DCM.

	**NMOSD**	**Long-segment DCM**	***p*-value**
Number of enhanced lesions	2 (1–12)	1 (1–1)	<0.05
Median number of the enhancement involved vertebral segments	3 (1–7)	1 (1–1)	<0.05
**Locations of the enhancement**			
C2	11 (40.7%)	1 (3.3%)	0.407
C3	14 (51.9%)	3 (10%)	0.222
C4	15 (55.6%)	9 (30%)	<0.05
C5	14 (51.9%)	26 (86.7%)	0.290
C6	13 (48.1%)	20 (66.7%)	<0.05
C7	6 (22.2%)	2 (6.7%)	<0.05
**Distribution of axial images**			0.115
White matter	9 (33.3%)	6 (20.0%)	
Gray and white matter	18 (66.7%)	24 (80.0%)	
**Enhancement pattern on sagittal images**			<0.05
Linear	10 (27.0%)	0	
Patchy	17 (63.0%)	0	
Transverse band (pancake-like)		15 (50%)	
Longitudinal oriented flake		15 (50%)	

NMOSD, neuromyelitis optica spectrum disorder; DCM, degenerative cervical myelopathy.

## Discussion

Differential diagnosis between NMOSD and DCM is not difficult under most circumstances. However, long-segment DCM with multi-level cord edema could present with LETM similar to NMOSD. The overlapped spectrum of clinical symptoms, including numbness, paresthesia, pain, and weakness, made it challenging to confirm the diagnosis. Additionally, patients with NMOSD may present with concurrent DCM, which made it difficult to differentiate the cause of the symptoms. Detailed analysis of MRI features is crucial for accurate and prompt diagnosis. Our study indicated that the enhancement of NMOSD patients and long-segment DCM patients differ significantly in number, length, location, and form.

From the perspective of the number and length of the enhanced lesion, our study presented similar results as previously reported. Multiple enhancements involving more than one vertebral level with various locations indicate NMOSD. Additionally, the enhancement involving the medulla oblongata is strongly indicative of NMOSD.

In the case of the enhancing pattern on the axial images, circumferential, peripheral, or scattered enhancements with sparing of the gray matter are frequently observed features for DCM (3, 9). In our study, circumferential enhancement involving only the white matter was specific for long-segment DCM. However, only a small number of cases showed this feature. The enhancement involving both the gray and white matter was noticed in most of the long-segment DCM cases. This might be explained by a more severe cord injury in long-segment DCM in comparison to the previously reported cohort involving a median number of two vertebral segments (3).

Regarding the form of the enhancement on sagittal images, the most frequently reported specific pattern of the spinal lesions in NMOSD was complete or incomplete ring-formed gadolinium enhancement surrounding the T1-weighted hypointense area (8). However, this feature was found in only one-third of the NMOSD patients (10). Other specific patterns were rarely described. In our study, linear enhancement was found in more than a quarter of NMOSD patients. None of the long-segment DCM cases have demonstrated a similar appearance. A previous study reported that linear formed T2WI hyperintense spinal cord lesion is a characteristic of NMOSD (11). Periependymal linear enhancement is a characteristic of the brain lesion of NMOSD (11, 12). A linear craniocaudal strip of enhancement located in the gray matter was reported to be specific for spontaneous spinal cord infarction (13). The linear enhancement described in our study was reported for the first time. It was located in the posterior column or anterior column of the white matter and could be discontinued as a dotted line. In long-segment DCM, a single transverse band appearing or pancake-like enhanced lesion on sagittal images immediately below the maximum spinal narrowing within the T2-weighted hyperintensity was reported to be strongly indicative of long-segment DCM (3, 9). In our study, one half of the long-segment DCM cases had a similar appearance as previously reported. The other half of the long-segment DCM cases had irregular flake-formed enhancement with a longitudinally oriented long axis. On the axial images, most of these lesions showed enhancement involving both white and gray matter.

The retrospective nature of this study made it impossible to assess the prevalence of gadolinium enhancement. Additionally, we were not able to follow the recruited cases to analyze the evolution of the enhancement pattern.

## Conclusion

By analyzing the number, length, location, and form of the gadolinium enhancements, NMOSD and long-segment DCM could be well-differentiated. Our study found that a linear formed enhancement and a single enhancement with a longitudinally oriented flake-like appearance located closely to the maximum spinal canal narrowing are evident for NMOSD and long-segment DCM, respectively.

## Data availability statement

The original contributions presented in the study are included in the article/supplementary material, further inquiries can be directed to the corresponding authors.

## Ethics statement

The studies involving human participants were reviewed and approved by Peking University Third Hospital Ethics Committee. Written informed consent from the patients or patients legal guardian/next of kin was not required to participate in this study in accordance with the national legislation and the institutional requirements.

## Author contributions

XS and QZ equally contributed to the acquisition, analysis, and interpretation of data for the work and writing of the manuscript. LZ and HY equally contributed to the conception or design of the work and revised the manuscript critically for important intellectual content. All authors approved the version to be published and agreed to be accountable for all aspects of the work in ensuring that questions related to the accuracy or integrity of any part of the work are appropriately investigated and resolved.
